# Detection of heteroresistance in *Mycobacterium tuberculosis* using nanopore-based amplicon sequencing and adaptive sampling

**DOI:** 10.1038/s41598-026-53258-0

**Published:** 2026-06-04

**Authors:** Diego A. Taquiri-Díaz, Diego M. Ramos-Lette, Omar A. Romero-Rodriguez, Carlos E. Barrios-Tapia, Carla A. Apaza-Quiroz, Benjamin Hurtado, Paul J. D. Avellaneda-Menéndez, Julio Orellana-Montes, Sonia Huaman, Jose L. Perez-Martinez, Katherine Vallejos-Sanchez, Candy León, Robert H. Gilman, Louis Grandjean, Mirko Zimic, Patricia Sheen

**Affiliations:** 1 Laboratorio de Bioinformática, Biología Molecular y Desarrollos Tecnológicos. Laboratorios de Investigación y Desarrollo, Facultad de Ciencias e Ingeniería, Universidad Peruana Cayetano Heredia, San Martín de Porres, Peru; 2 Department of International Health. Bloomberg School of Public Health, Johns Hopkins University, Baltimore, USA; 3 University College London, London, UK

**Keywords:** Tuberculosis, Mixed infections, Heteroresistance, Nanopore, Amplicon Sequencing, AS, Computational biology and bioinformatics, Diseases, Genetics, Microbiology

## Abstract

Heteroresistance (HR) in *Mycobacterium tuberculosis*—the coexistence of drug-susceptible and drug-resistant subpopulations within the same host—poses a major obstacle to effective tuberculosis (TB) diagnosis, treatment, and control. The primary objective of this study was to determine whether Oxford Nanopore Technologies (ONT)-based workflows could accurately detect heteroresistance and compare their performance. To achieve this, we first optimized ONT sequencing workflows using cultured isolates and TB-positive clinical sputum, identifying phenol-based DNA extraction with ligation library as the best-performing combination, though Kit-Ligation had the best performance in amplicon sequencing from culture. We then evaluated two enrichment strategies—amplicon sequencing and Adaptive sampling (AS)—using synthetic mixtures of drug-susceptible (H37Rv) and drug-resistant (DM97) strains at defined ratios. Amplicon sequencing achieved coverage > 4,000× across target genes and reliably detected resistant alleles at frequencies as low as 1%. AS enriched 14 resistance loci with ~ 3-fold higher on-target depth compared to non-enriched nanopore whole-genome sequencing (WGS), enabling accurate detection of resistant subpopulations at 5% while retaining genomic context for lineage assignment. Together, these results demonstrate that, among the ONT workflows evaluated, nanopore amplicon sequencing provides superior sensitivity, whereas AS offers an intermediate approach, increasing depth while retaining some genomic context. These complementary approaches represent scalable strategies that could enhance TB heteroresistance detection and surveillance in high-burden settings.

**Supplementary Information:**

The online version contains supplementary material available at 10.1038/s41598-026-53258-0.

## Introduction

Tuberculosis (TB), caused by *Mycobacterium tuberculosis*, continues to be one of the leading causes of infectious disease-related morbidity and mortality worldwide. In 2023 alone, approximately 10.8 million individuals fell ill with TB, and 1.25 million died from the disease^1^. Although TB is curable and preventable, the emergence of drug-resistant forms of the disease has become a critical challenge in global health. Multidrug-resistant TB (MDR-TB) and extensively drug-resistant TB (XDR-TB) represent significant obstacles to achieving TB control, particularly in high-burden countries with limited diagnostic infrastructure^2^. Although drug-resistant TB comprises only about 5% of total cases, it is responsible for up to 20% of TB-related deaths^3^. The COVID-19 pandemic has further exacerbated these challenges by disrupting diagnosis and treatment efforts. In Peru, for instance, TB cases increased by 8.5% and TB-related morbidity rose by 6.7% from 2021 to 2022, and treatment failure was associated with an alarmingly high ~ 50% mortality rate despite an ~ 85% effective treatment regimen^4^.

The early and accurate detection of drug resistance is essential for initiating appropriate therapy and improving treatment outcomes^5^. Traditionally, resistance has been assessed using phenotypic drug susceptibility testing (DST), which is slow and requires biosafety-level 3 laboratory conditions^6^. More recently, molecular diagnostic tools such as the GeneXpert MTB/RIF and line probe assays (LPAs) have revolutionized TB diagnosis by enabling rapid identification of rifampicin resistance and other mutations. However, these assays often miss minority variants present below a certain frequency threshold and are limited to detecting known mutations within predefined regions^7^.

A significant challenge in tuberculosis control is heteroresistance (HR), the coexistence of drug-susceptible and drug-resistant *M. tuberculosis* populations within a single host. HR can arise through at least two mechanisms: mixed infection, in which a patient is concurrently infected with genetically distinct strains that may differ in drug-susceptibility profiles^8,9^, and within-host clonal evolution, in which resistance-conferring mutations emerge under antibiotic pressure^9^. Regardless of its origin, HR leads to the underdetection of resistance and subsequent treatment failure^8,10^. Notably, identifying these resistant subpopulations is significantly more complex for genomic-based predictions than for phenotypic methods. While phenotypic drug susceptibility testing (DST) naturally identifies resistance through the growth of resistant bacilli, genomic approaches must accurately distinguish low-frequency variants from sequencing artifacts, requiring high depth of coverage and specialized bioinformatic pipelines.

Heteroresistance has been reported in approximately 5–10% of clinical TB cases, although this estimate likely oversimplifies a highly variable phenomenon influenced by drug type, diagnostic approach, detection thresholds, and population characteristics^3,11^. It is also associated with poor treatment outcomes, relapse, and the emergence of full-blown resistance^12^. Its global occurrence, documented in high-burden countries such as Peru^13^, Uzbekistan^9^, England^14^, and India^15^, underscores its epidemiological and clinical relevance^16^.

However, current frontline diagnostics have difficulty detecting these mixed infections and minor resistant subpopulations. While the traditional GeneXpert MTB/RIF assay lacks the sensitivity to detect heteroresistance when a resistant subpopulation falls below 60% of the total^17^, newer iterations like GeneXpert Ultra and line probe assays (LPAs) have shown variable limits of detection ranging from 20 to 70% and 5–10% respectively, depending on the specific *rpoB* mutation present^17,18^. However, these thresholds remain significantly higher than the 1% sensitivity achieved by standard phenotypic methods. Phenotypic methods can detect resistant subpopulations, but their performance depends on the platform used. The classical solid-media proportion method can detect resistant bacilli at frequencies as low as 1%, whereas liquid culture-based phenotypic DST (e.g., BACTEC MGIT 960) is faster—typically providing results within 10–14 days compared to several weeks for solid media—and is widely used in routine diagnostics, although it remains culture-dependent and its sensitivity for detecting low-frequency heteroresistance may vary depending on the drug and mutation^19–21^. Microscopy-based assays such as MODS offer lower-cost alternatives, but they rely on subjective interpretation^22,23^. Genotyping methods used for epidemiological surveillance (e.g., MIRU-VNTR, spoligotyping, IS6110-RFLP) also lack the resolution to identify minority variants within an infection^24–27^.

Whole-genome sequencing (WGS) has emerged as a powerful approach for detecting drug resistance, including heteroresistance, by enabling comprehensive assessment of all resistance-associated genes^28–30^. Untargeted WGS of clinical samples may be inefficient due to limited coverage of resistance-relevant loci, particularly in direct sputum specimens with abundant host DNA^31^. Targeted sequencing approaches, such as the Deeplex Myc-TB amplicon assay, overcome this limitation by amplifying known resistance genes, thereby improving sensitivity and cost-efficiency^32^.

Nanopore sequencing provides additional unique advantages: Real-time data acquisition, high throughput, portability, and recently improved base-calling accuracy (< 6% error rates)^33^. In this context, Oxford Nanopore Technologies (ONT) sequencing offers a promising alternative due to its long-read capacity, real-time analysis, and ability to operate in low-resource environments. Furthermore, Oxford Nanopore’s Adaptive sampling (AS) technology enables real-time enrichment of specific genomic regions without additional wet-lab steps, significantly enhancing the detection of target sequences in clinical specimens. Notably, AS works by dynamically accepting or rejecting DNA fragments during sequencing, achieving targeted depth of coverage at resistance-associated loci without extra laboratory procedures. This strategy is particularly advantageous for rapid diagnostics in low-resource or decentralized settings^34^.

In this study, we pursued three primary objectives to establish a robust framework for tuberculosis (TB) resistance detection using Oxford Nanopore Technologies (ONT), as detailed in Supplementary Fig. S1. First, we optimized sequencing performance across both clinical sputum and cultured isolates by benchmarking key variables, including DNA extraction methods (Phenol-Chloroform vs. kit-based), DNA input types (WGS vs. amplicon), and library preparation strategies (Ligation vs. Rapid). This stage utilized sequencing performance metrics—total output (Gb), read length (N50), and mean depth—to identify the best-performing workflows for each sample type. Second, we established the analytical limit of detection (LoD) for heteroresistance using targeted amplicon sequencing of whole-cell H37Rv/DM97 mixtures spiked into clinical sputum or PBS matrices. Third, we determined the LoD for Adaptive Sampling (AS) using purified high-molecular-weight DNA mixtures of the same strains. By comparing these targeted enrichment strategies against non-enriched whole-genome sequencing (WGS), we aimed to define the optimal balance between high-sensitivity minority variant detection and comprehensive genomic surveillance.

## Results

### Comparison of DNA extraction methods and amplicon generation

We evaluated two DNA extraction protocols Phenol and Kit for their ability to produce high-quality DNA suitable for downstream nanopore sequencing across a set of 33 clinical sputum samples. Qubit fluorometric quantification demonstrated that the Phenol method yielded a higher median DNA concentration of 99.6 ng/µL (IQR: 35.2–168.0 ng/µL) compared to 65.2 ng/µL (IQR: 52.2–102.0 ng/µL) for the Kit method (Supplementary Fig. S2; Supplementary Table S1). The Phenol method also achieved higher maximum DNA recovery, with yields up to 58.4 µg, whereas the Kit method reached up to 11.0 µg (Supplementary Table S1). However, TapeStation analysis (Supplementary Fig. S2) indicated that the Kit method produced longer DNA fragments with a median of 15.6 kb (IQR: 14.6–16.4 kb), compared with 7.6 kb (IQR: 6.8–8.9 kb) for the Phenol method. Together, these findings indicate a trade-off between DNA yield and fragment length across the two extraction methods.

Following the extraction assessment, a representative subset of 12 clinical sputum samples, together with 12 cultured isolates, was selected for subsequent workflow optimization. Using DNA from this subset, we successfully amplified all 14 targeted resistance-associated genes, arranged in three primer pools. All expected PCR amplicons (ranging from ~ 876 bp to 3662 bp in size) were visible by agarose gel electrophoresis. Notably, the Kit method produced visibly brighter and more robust amplicon bands compared to the Phenol extracts (Supplementary Fig. S3). Additionally, for the Phenol-extracted samples (Supplementary Fig. S3), brightly fluorescent material was retained in the loading wells. Because of its massive size (mean N50 > 7 kb) and high abundance, the intact genomic template DNA preserved by the phenol-chloroform extraction can fail to migrate into the agarose gel matrix alongside the much smaller targeted amplicons, remaining trapped in the wells. Because standardized 50 ng DNA inputs were used across all PCR assays, this difference is not due to input quantity. Instead, the improved amplification efficiency is likely driven by differences in DNA purity and PCR compatibility, as column-based extraction may more effectively remove residual inhibitors (e.g., trace phenol or salts) that can interfere with amplification of larger targets. Serial dilutions of the input DNA indicated that robust amplification was achievable with as little as 0.001 ng/µL of template DNA. Notably, Primer Pool 1, which included several high-GC-content genes (e.g., *rpoB*, *katG*), required minor adjustments to annealing temperatures to achieve optimal yields.

### Comparison of nanopore sequencing methods

Across flow cell runs, we generated a total of approximately 58 million reads. The mean read-length N50 was 4.1 kb (range: 409 kb-8391 kb), and the median per-read quality score (Q-score) was 18.34 (range: 10.5–19.9).

**Fig. 1:**
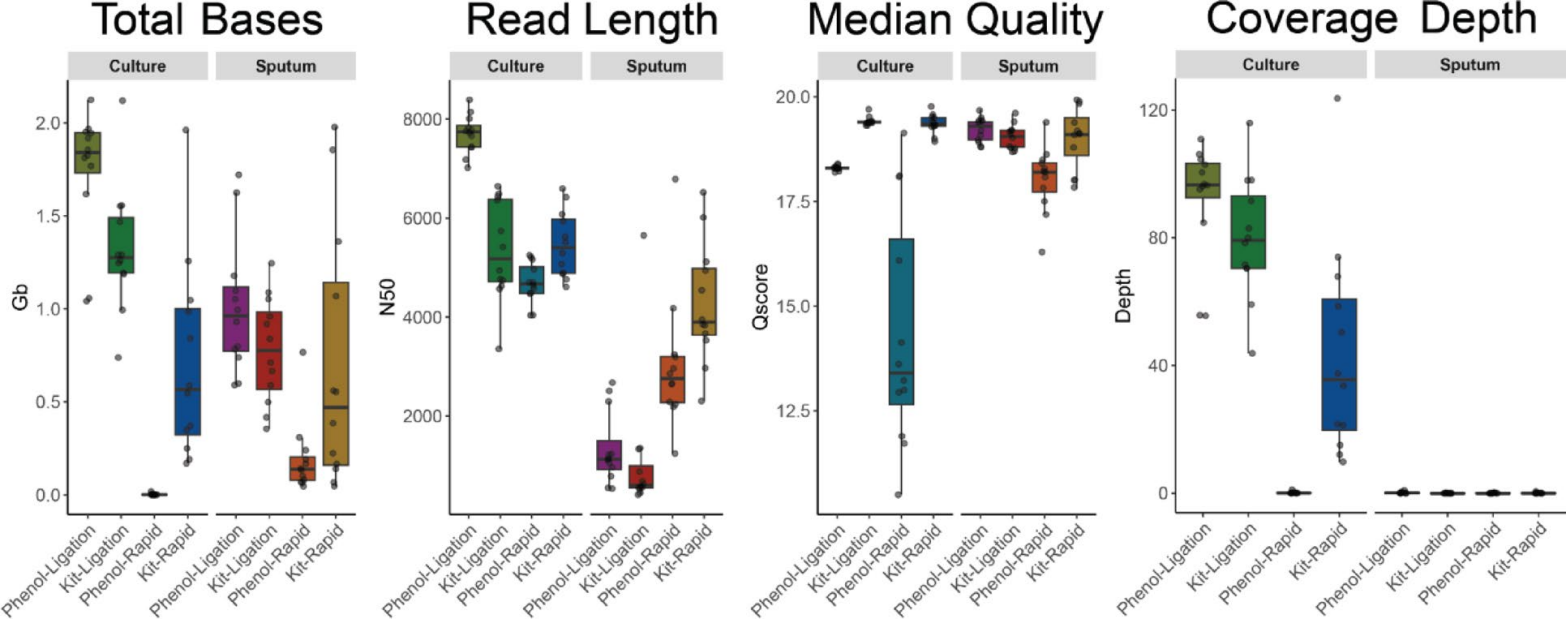
Sequencing performance and resistance gene coverage in clinical TB samples using nanopore whole-genome sequencing. Boxplots of sequencing performance metrics – total bases (Gb), read length (N50), median Q-score and Depth – for sputum and culture samples processed using either ligation-based or rapid library preparation in combination with kit or phenol-based DNA extraction. Boxplots of the mean depth across the *M. tuberculosis* genome in samples processed with each combination of library preparation (ligation vs rapid) and DNA extraction technique (kit vs phenol). All data are derived from whole-genome sequencing of clinical TB patient samples, including both cultured isolates and direct sputum samples. Note: Labels ‘Kit-Ligation’ and ‘Kit-Rapid’ in the figures refer to the Zymo-based extraction protocol using the Quick-DNA™ Miniprep Plus (Zymo Research).

In cultured isolate samples, when using whole genome sequencing, the Phenol-Ligation method showed the strongest overall sequencing performance, with an average output of 1.74 Gb ± 0.35 Gb per barcode (compared to 1.32 Gb ± 0.34 Gb from the second best method, Kit-Ligation) and the longest read lengths, around N50 of 7.7 kb (IQR: 7.4–7.9 kb) (compared to a median of 5.4 kb [IQR: 4.9–6.0 kb] from the second best method, Rapid-Kit) (Fig. 1). However, the median quality score was slightly lower at 18.3 (IQR: 18.3–18.3). In contrast, the rapid library groups on average consistently yielded the lowest total bases (0.36 Gb ± 0.52 Gb), shortest read lengths (N50 median of 4.9 kb [IQR: 4.6–5.3 kb]), and poorest quality scores (median of 18.9 [IQR: 13.5–19.3]). In terms of sequencing depth, the Phenol-Ligation method achieved a mean depth of around 92.11× (± 18.25 × SD) across the entire *M. tuberculosis* genome, using 12 culture samples sequenced on a single MinION flow cell (Fig. 1).

Among the methods tested for TB detection from sputum samples, the Phenol-Ligation approach delivered the best overall performance. It achieved a median output of 0.96 Gb per barcode (IQR: 0.77–1.12 Gb) per barcode, allowing efficient sequencing of 12 samples on a single flow cell, with a median Q-score of 19.3 (IQR: 19.0–19.4) and a read length (N50) of 1.1 kb (IQR: 0.9–1.5 kb) (Fig. 1). In contrast, the Phenol-Rapid method showed the poorest performance, with a low mean total output of 0.19 Gb (± 0.20 Gb SD) and a median Q-score of 18.2 (IQR: 17.7–18.4), though it unexpectedly produced the second longest reads, with a median N50 of 2.8 kb (IQR: 2.3–3.2 kb) among sputum samples. Notably, the Phenol-Ligation method also yielded the highest *M. tuberculosis* read count (Supplementary Fig. S4). While this is sufficient to confidently detect the presence of TB, it remains inadequate for resistance profiling, as the mean coverage per resistance gene is only 0.18× (± 0.26 × SD) (Fig. 1), whereas at least 10× coverage per gene is generally needed for reliable resistance calling.

**Fig. 2:**
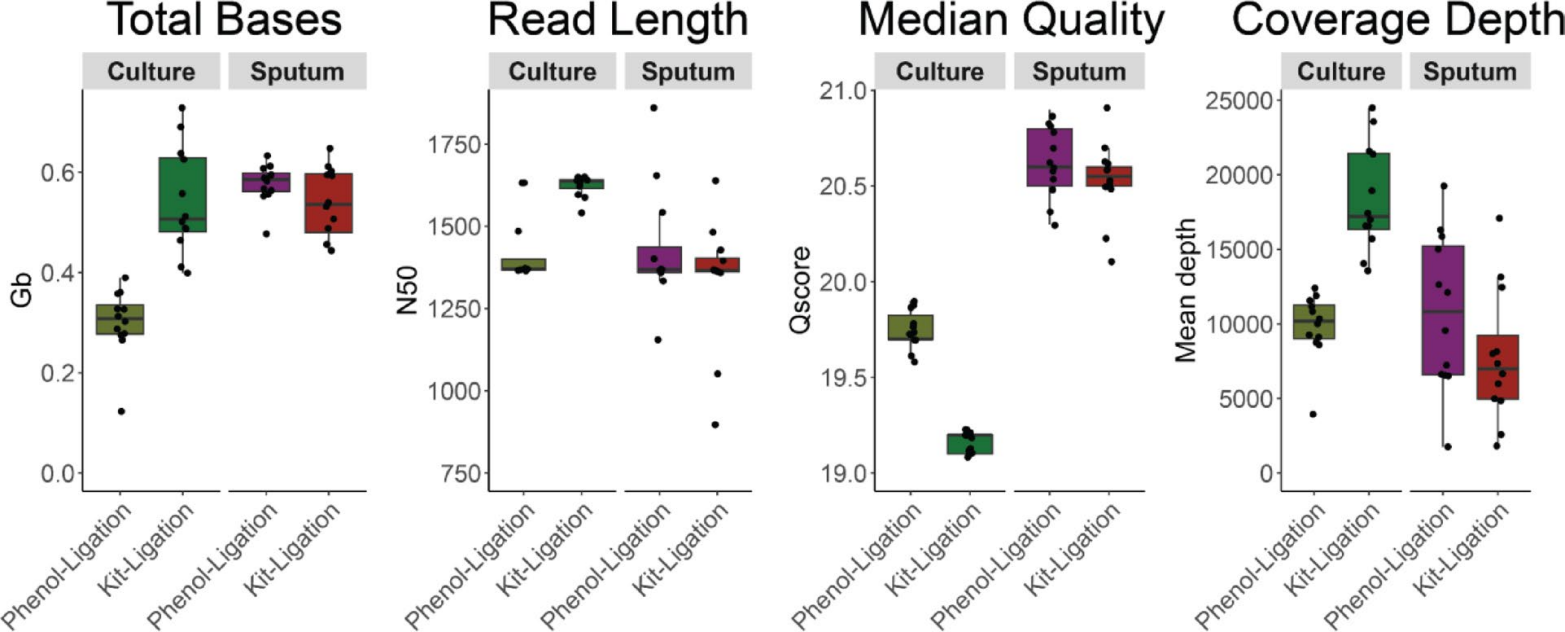
Sequencing performance and resistance gene coverage in clinical TB samples using nanopore amplicon sequencing. Initial experiments were performed using a 14-gene amplicon panel; a subsequent targeted sequencing run focused only on four genes (*inhA*, *katG*, *pncA*, and *rpoB*). Sequencing metrics comparing kit-based vs. phenol-based DNA extraction for sputum and culture samples (14-gene panel).

To compare amplicon-based sequencing methods in sputum samples, we found that the Phenol-Ligation method demonstrated superior performance compared to other approaches. This method achieved the highest output, averaging 0.58 Gb ± 0.04 Gb per sample when sequencing 12 samples simultaneously on a single MinION flow cell, along with optimal read length with a median N50 of 1.4 kb (IQR: 1.4–1.4 kb) and quality scores with a median Q-score of 20.6 (IQR: 20.5–20.8) (Fig. 2). The Phenol-Ligation method also yielded the highest median depth across antimicrobial resistance (AMR) genes, reaching 14,228× (IQR: 9,102–19,379×) (Supplementary Fig. S5). However, coverage varied significantly between individual resistance genes; for example, in sputum Phenol-Ligation amplicon runs, the *eis* gene had a median depth of 41,336x (IQR: 34,039–50,500x), whereas *rpoB* achieved only about 737× (IQR: 58–1,246x) (Fig. 2). Further analysis revealed that this variability was significantly correlated with amplicon target size (R²=0.285, p-value < 0.001), indicating that larger genes such as *rpoB* tended to have considerably lower coverage depth in sputum (Fig. S6) and culture (Supplementary Fig. S7).

To address the reduced coverage observed for larger targets such as *rpoB* in the 14-gene panel, and to assess whether this limitation was driven by multiplexing complexity, we performed a focused amplicon sequencing experiment. Specifically, we reduced the primer pool to four key resistance-associated genes—*rpoB* (rifampicin resistance), (pyrazinamide resistance), *inhA* (isoniazid resistance), and *katG* (isoniazid resistance)—to test whether a simplified panel could recover the lost depth. Using this approach, we achieved a median coverage depth of approximately 5,614× (IQR: 2,150–9,606×) across 12 sputum samples sequenced on a single MinION flow cell. The longest gene, *rpoB*, attained a median coverage depth per 1000 pore-hours of 789× (IQR: 636–949×) (Table 1). To enable direct comparison across library preparation methods, we normalized sequencing depth per 1,000 pore-hours (kPH). This metric corrects for potential differences in flow cell performance and run duration. This improved sequencing depth significantly enhances the detection capability for heteroresistance, particularly critical for rifampicin resistance.

In addition to sputum-based assays, we evaluated amplicon sequencing performance on *M. tuberculosis* culture samples. For these clean, non-viscous matrices, the Kit-Ligation proved to be the most effective method. Sequencing of cultured isolates using this kit yielded a consistent output of approximately 0.54 Gb ± 0.11 Gb per sample, with a read length N50 of 1.6 kb (IQR: 1.6–1.6 kb) and a median quality score of 19.2 (IQR: 19.1–19.2) (Fig. 2).

**Table 1:** Normalized coverage depth per 1 000 pore-hours (× kPH⁻¹) for the targeted four-gene panel in sputum samples using phenol-based extraction, comparing ligation-based vs. rapid library preparation methods. Values represent median depth and interquartile ranges (Q1–Q3).

Gene	Phenol-Ligation *N* = 12	Phenol-Rapid *N* = 12
*inhA*	3611 (2904, 4288)	430 (250, 711)
*katG*	3471 (2925, 4361)	391 (227, 632)
*pncA*	2746 (2267, 3163)	165 (88,216)
*rpoB*	789 (636, 949)	295 (162,463)

### Generation of synthetic heteroresistant mixtures

We prepared defined mixtures of H37Rv (susceptible) and DM97 (resistant) *M. tuberculosis* strains to represent resistant subpopulation frequencies from 1% up to 99%. Proportion-based phenotypic drug susceptibility testing on 7H11 agar confirmed that rifampicin, isoniazid and pyrazinamide resistance could be detected by culture in these mixtures at levels as low as ~ 1%. For example, as shown in Supplementary Fig. S8, even the mixture with only 1% DM97 produced visible colony growth on isoniazid-containing media, validating the sensitivity of the reference phenotypic method.

These synthetic mixtures served as controlled reference standards for evaluating the sequencing-based methods. We used them to benchmark the sensitivity of nanopore amplicon sequencing in detecting low-frequency resistance mutations under controlled conditions.

### Genotypic characterization of DM97 multi-drug resistant reference strain

The DM97 clinical strain has been extensively utilized as a multi-drug resistant TB control since the late 1990s for various laboratory studies and procedures. Despite its longstanding use, detailed genotypic information about DM97 has remained unavailable, underscoring the importance of conducting comprehensive genomic sequencing to fill this gap.

Genotypic typing through spoligotyping confirmed that the DM97 isolate belongs to lineage 4 (T), which is commonly observed in Peru. D*e novo* assembly of DM97 required manual curation due to the presence of contaminant sequences. Only a 4,408,907 bp circularized contig was retained. The final assembly was determined to be complete (100.0% completeness, 0.77% contamination), with a GC content of 66% and an overall coding density of 90.4%. In total, 4,070 coding sequences were predicted, with an average gene length of 327.39 amino acids.

Whole genome sequencing allowed direct resistance genotyping of DM97, identifying mutations in the *rpoB*, *katG*, *pncA*, and *embB* genes (Table 2). It harbored a mutation in *rpoB* (p.S450L) associated with rifampicin resistance, *katG* (p.S315T) associated with isoniazid resistance, *embB* (p.D354A) associated with ethambutol resistance, and *pncA* (p.T135P) associated with pyrazinamide resistance.

**Table 2:** Key resistance-associated mutations identified in *M. tuberculosis* DM97.

Genome position	Locus tag	Gene name	Variant	Drug
761,155	Rv0667	*rpoB*	S450L	Rifampicin
2,155,168	Rv1908c	*katG*	S315T	Isoniazid
2,288,839	Rv2043c	*pncA*	T135P	Pyrazinamide
4,247,574	Rv3795	*embB*	D354A	Ethambutol

### High coverage and low LoD in amplicon sequencing

Nonparametric estimates of the limit of blank (LoB) were calculated for each method (Table 3). Amplicon sequencing showed the lowest LoB values (0.42–0.46%), supporting reliable detection of resistant alleles down to 1% in both sputum and non-sputum samples and providing a practical baseline for distinguishing true low-frequency variants from background noise. Results from amplicon sequencing were broadly consistent with AS across the shared target genes, with the exception of the *embB* p.D354A variant, which was only detected by AS as it was not included in the amplicon panel.

**Fig. 3:**
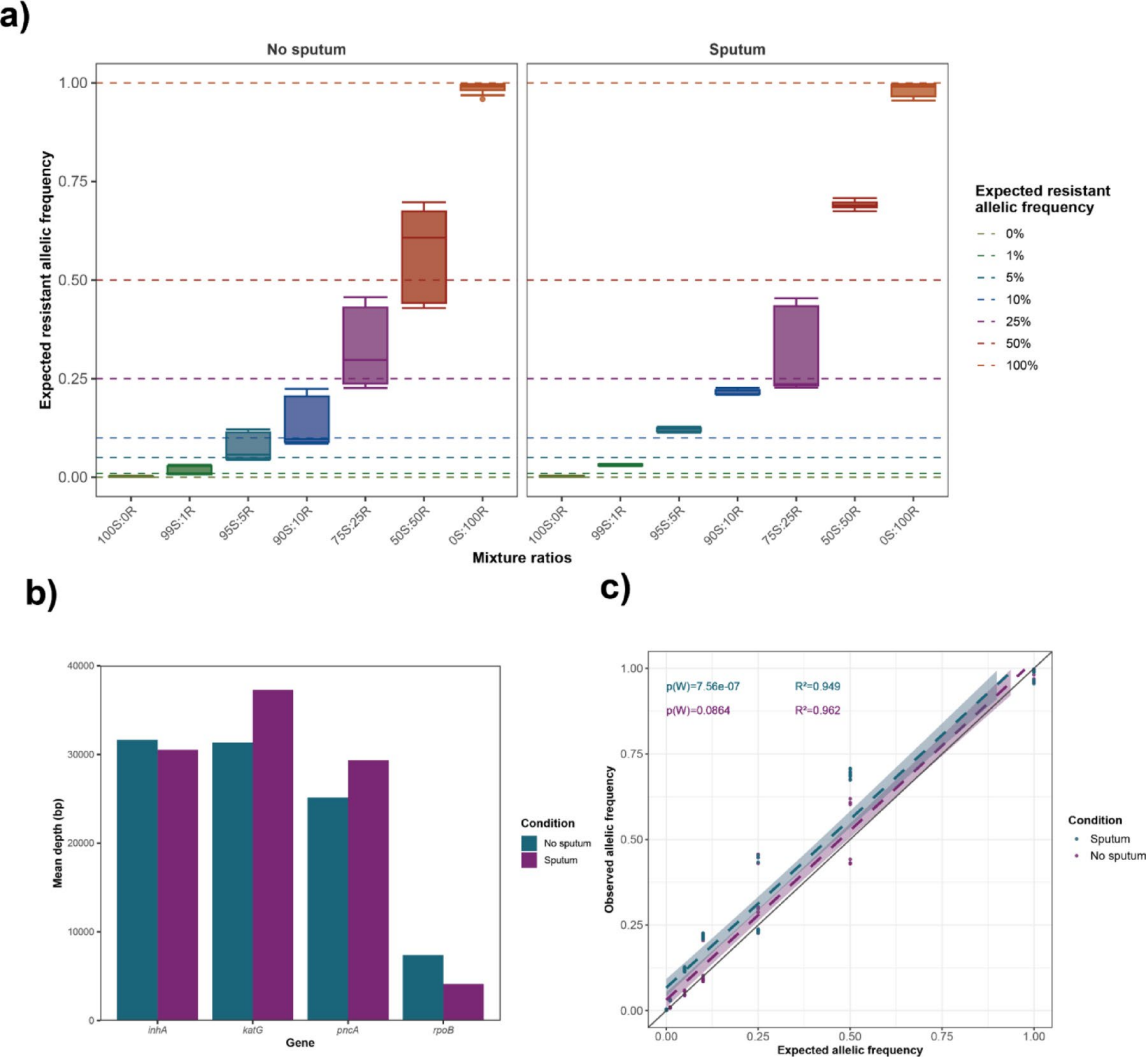
Evaluation of the limit of detection for mutant alleles in a resistant strain using targeted amplicon sequencing of heteroresistant *M. tuberculosis* mixtures, sequenced with and without a sputum matrix. (**a**) Observed mutant allelic frequency for three resistance-associated mutations (*katG* S315T, *pncA* T135P, *rpoB* S450L) across mixtures containing varying proportions of sensitive and resistant bacilli. Mixture labels (e.g., 99 S:1R) indicate the percentage of Sensitive (S) to Resistant (R) strains (e.g., 99 S:1R = 99% sensitive, 1% resistant). Dashed lines mark the expected mutant proportion in each mixture. For each mixture, three variants were evaluated across three biological replicates (*n* = 9 total measurements per condition). (**b**) Mean sequencing depth across four PCR-amplified resistance-associated genes (*inhA*,* pncA*,* katG*,* rpoB*), comparing sputum and non-sputum conditions. (**c**) Concordance between expected and observed mutant allelic frequency across all mixtures; the red dashed line is the identity (y = x). Symbols distinguish variants and colour distinguishes preparation method.

Amplicon sequencing yielded exceptionally high coverage across all targeted loci, with mean depths consistently exceeding 4,000×: *rpoB* (7,370× in non-sputum and 4,117× in sputum), *inhA* (31,648× and 30,540×), *katG* (31,338× and 37,283×), and *pncA* (25,139× and 29,352×) (Fig. 3b). Amplicon sequencing accurately recovered resistant allele frequencies across synthetic mixtures with and without sputum. Figure 3a shows reproducible detection of variants down to 1% in both conditions, consistent with the LOD reported in Table 3. Interestingly, boxplots were longer for no sputum samples, indicating greater spread in allele frequency estimates compared to sputum mixtures.

Regression of observed versus expected frequencies (Fig. 3c) confirmed strong linearity for both approaches (R² = 0.962 non-sputum; R² = 0.949 sputum). While non-sputum samples showed no significant deviation from the 1:1 line (*p* = 0.086), sputum samples displayed a significant deviation (*p* = 7.56 × 10⁻⁷). These results indicate that sputum introduces modest matrix effects over allele frequency estimates, and non-sputum samples are more variable across replicates, but both approaches remain robust for rare variant detection, including resistant alleles at 1%.

### AS enhances coverage with comparable detection limits to WGS

**Fig. 4:**
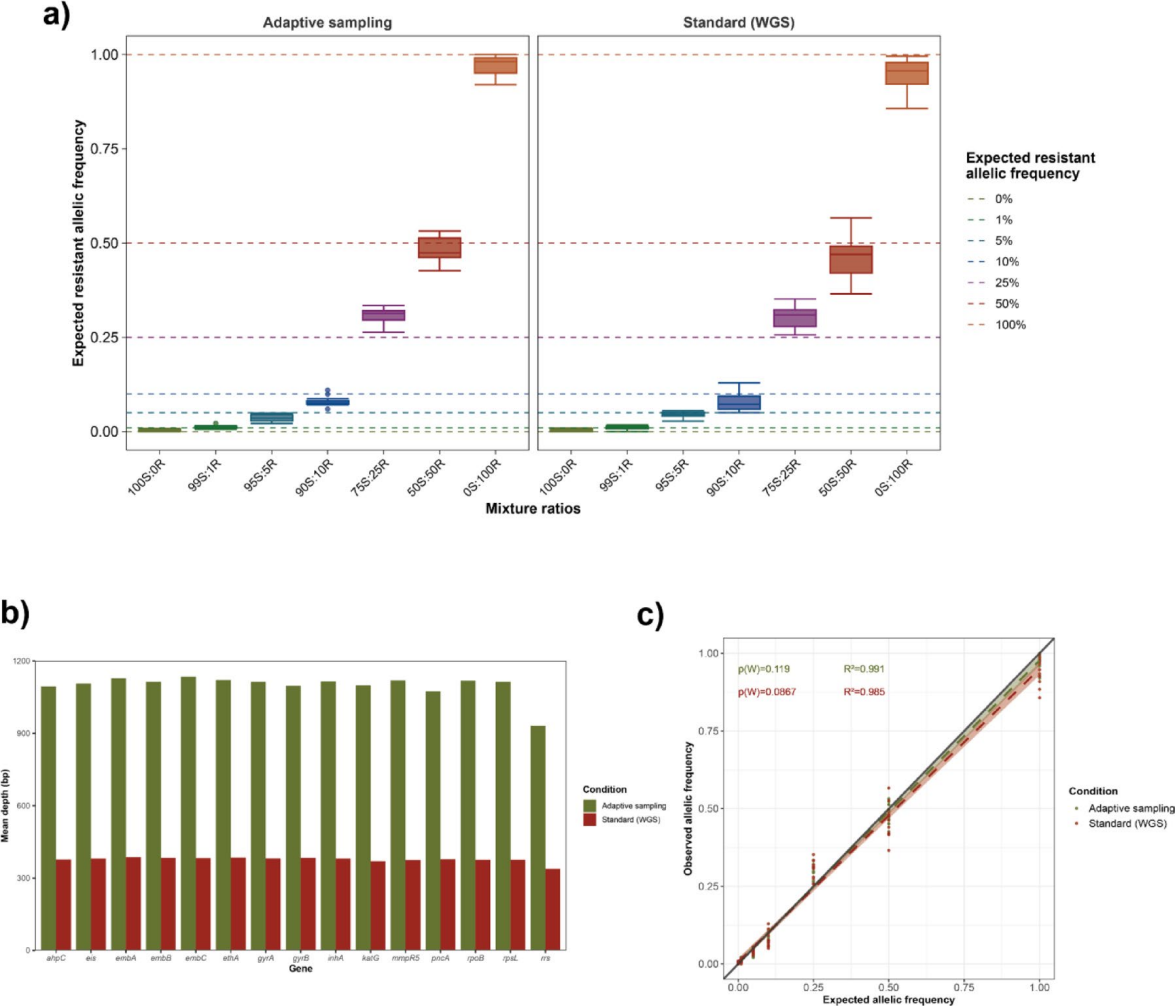
Evaluation of limit of detection of AS for detecting resistance in heteroresistant *M. tuberculosis* samples. (**a**) Boxplots of the detected resistance allele percentage in heteroresistant mixtures for the first experimental run, which included mixtures ranging from 100% down to 0% resistant DM97 strain. Results are shown separately for AS and Standard sequencing. Mixture labels in the x-axis indicate the ratio of the resistant DM97 strain (R) to the susceptible H37Rv strain (S). AF: Allelic frequency. For each mixture, four variants (*katG* S315T, *pncA* T135P, *rpoB* S450L, *embB* D354A) were evaluated across three biological replicates (*n* = 12 total measurements per condition). (**b**) Bar chart comparing the mean read depth achieved by AS vs. Standard sequencing for the target resistance genes and spoligotype loci. (**c**) Scatter plot of expected versus observed allelic frequencies for four resistance‑associated variants across different mixture proportions. Points are colour‑coded by sequencing strategy, purple for AS and teal for Standard sequencing.

AS runs achieved an average on-target coverage of 996.75× for the specified resistance genes and spoligotyping loci (Supplemetary Fig. S9), based on the exact per-gene values [*gyrB gyrA* (1006.01×), *rpoB mmpR5 rpsL* (1052.73×), *rrs* (952.10×), *inhA* (990.21×), *katG* (994.16×), *pncA* (971.04×), *eis oxyR*-*ahpC* (1020.28×), spacer43 spacer1 (948.78×), *embC embA embB* (1041.85×), and *ethA* (990.38×)]. In contrast, the standard method reached an average coverage of 345.39× [*gyrB gyrA* (349.03×), *rpoB mmpR5 rpsL* (350.17×), *rrs* (333.30×), *inhA* (345.69×), *katG* (340.70×), *pncA* (342.78×), *eis oxy-R-ahpC* (346.90×), spacer43 spacer1 (343.50×), *embC embA embB* (351.47×), and *ethA* (350.35×)]. On-target depth in AS exceeded that of the standard approach by 2.89-fold (Fig. 4b). Given that AS and standard were run simultaneously on the same PromethION flow cell using a half-pore split, this 2.89-fold enrichment represents a direct within-flow-cell comparison. Additionally, AS achieved ~ 91× mean genome-wide coverage (off-target) (Supplementary Fig. S9).

AS and non-enriched WGS showed high LoB values (0.84% and 0.93%, respectively) (Table 3). Both provided accurate estimates of resistant allele frequencies across synthetic mixtures. Figure 4a shows that variants at ≥ 5% were consistently recovered by both approaches, in agreement with the calculated limit of detection of 5% (Table 3). Neither method reliably detected resistant alleles at 1%. Boxplots revealed comparable performance, however WGS displayed slightly broader variability at intermediate allele fractions. Regression of observed versus expected frequencies (Fig. 4c) confirmed linearity (R² = 0.991 AS ; R² = 0.985 WGS) without significant deviation from expectation (*p* = 0.119 AS; *p* = 0.0867 WGS).

We estimated allele frequencies from the read pile-up—that is, per-position counts of bases in the alignments—yielding the most precise estimates across all mixture ratios and outperforming automated variant callers. When run via TB-Profiler, BCFtools failed to detect resistant subpopulations below ~ 25%, missing low-frequency variants in our dataset (Supplementary Fig. S10).

**Table 3:** Performance metrics of nanopore enrichment methods for heteroresistance detection.

Method	Limit of blank (%)	Limit of detection (%)
Amplicon (Sputum) *N* = 9	0.417	1
Amplicon (No sputum) *N* = 9	0.462	1
AS *N* = 12	0.840	5
Standard Nanopore WGS (non-enriched) *N* = 12	0.926	5

Amplicon sequencing proved more sensitivity than AS for these low-frequency variants, owing to the much higher sequencing depth achieved per locus. However, AS provided broader genomic context: beyond the targeted drug-resistance loci, it enabled whole genome coverage and allowed lineage determination through genome-wide SNP analysis—capabilities not offered by the amplicon method (Supplementary Fig. S9).

### Amplicon sequencing and AS

These combined results demonstrate that all four strategies (AS, standard non-enriched nanopore WGS, amplicon with sputum, amplicon without sputum) reliably detect and quantify low-frequency resistance variants in mixed-strain *M. tuberculosis* samples.

Both nanopore enrichment strategies showed a high concordance in detecting known resistance variants across samples. However, each method offered distinct advantages. Amplicon sequencing required less input DNA and achieved greater sensitivity for low-frequency resistance alleles, particularly in the controlled synthetic mixtures. AS, by contrast, provided much broader genomic coverage, enabling simultaneous resistance profiling and lineage analysis from the same run.

Notably, AS achieved its enrichment without any prior PCR amplification or additional laboratory processing, making it a highly scalable option in clinical settings where rapid turnaround is critical. In practice, the two approaches can be combined as complementary strategies—leveraging the high sensitivity of amplicon sequencing alongside the comprehensive analysis afforded by AS—to improve heteroresistance detection and whole-genome characterization of *M. tuberculosis* strains.

## Discussion

Our study demonstrates the potential of ONT sequencing for detecting heteroresistance in *M. tuberculosis*. In particular, our AS strategy proved value for increasing read depth over regions of interest, even in low DNA mixtures; evaluating AS directly on low bacteria load samples remains to be tested. This is advantageous for the reliable detection of minority variants and supports previous reports on the utility of AS for real-time target enrichment^35^. In this application, the main value of ONT was not long-read sequencing alone, but its ability to support flexible targeted workflows: high-depth amplicon sequencing for sensitive minority-variant detection and adaptive sampling for enrichment of resistance-associated loci while retaining genomic context.

During library preparation, ligation libraries generally delivered higher yields and longer reads than rapid kits in our dataset, consistent with previous reports^36^. However, performance varied across sample types and experimental conditions, and variability across replicates was observed. Rapid kits, although trading depth for speed (< 2 h bench time), may still be useful when reduced hands-on preparation and workflow simplicity are priorities. Using R10.4.1 flow cells and Dorado 0.7 SUP, we achieved median Q-scores close to 20 across both WGS and amplicon sequencing, in line with recent multicentre data^37^. A single R10.4.1 flow cell provided median coverage of ~ 4 000 × across the four genes *rpoB*, *katG*, *inhA* and *pncA*. This depth enabled detection of *rpoB* S450L at 1% allele frequency in H37Rv: DM97 mixtures, performance equivalent to culture and clearly superior to GeneXpert Ultra’s ~ 10% limit of detection^17^. AS, in turn, reached ~ 1 000 × mean depth for fourteen resistance loci while retaining > 90 × genome-wide coverage (Supplementary Fig.), and called 5% mutations, parallel findings reported similar values previously^34,38^. Whole-genome sequencing (WGS) supplies complete genomic context, revealing mixed infections and novel resistance variants that targeted panels overlook. While some studies suggest culture steps can purge minority clones^39^, the degree to which culturing constrains genetic diversity remains a matter of debate. Recent evidence suggests that when bioinformatic artifacts are accounted for, culture-based sequencing can accurately mirror the diversity found in diagnostic sputum samples^40^. Nonetheless, direct sequencing remains critical for rapid diagnostics and for detecting variants with significant fitness costs that may be underrepresented in liquid media.

Consistent and adequate coverage across high-GC loci such as *rpoB* and *katG* mirrors the performance reported by Gómez-González^38^, who demonstrated Nanopore’s superiority over short-read platforms in these regions; nevertheless, we observed sporadic PCR drop-outs, echoing the variability noted previously^41^.

Head-to-head comparisons highlight the residual sensitivity gap across diagnostic platforms. Conventional GeneXpert flags “RIF-R” only when ≥ 60% of bacilli are resistant^17^, and line-probe assays (LPA) visualise mutant bands above ~ 5%. Ultra-sensitive tools such as ddPCR lower the LoD to < 1%, but their single-gene focus limits scope. Deep-amplicon sequencing (Illumina Deeplex Myc-TB) merges a 1–3% LoD with simultaneous interrogation of up to 24 genes, approaching the phenotypic gold standard^41,42^. Our results corroborate this: the 5:95 DM97:H37Rv mixture was called resistant by amplicons and AS. The read pile-up clearly showed the mutations, whereas automated callers such as TB-Profiler (BCFtools) missed variants below 25%, likely due to conservative filtering, despite ground-truth confirmation from deep DM97 sequencing.

The detection of low-frequency variants is inherently limited by both the sequencing platform’s error profile and the sensitivity of the bioinformatic ‘caller.’ While Oxford Nanopore technology historically faced challenges with high raw error rates, the transition to R10.4.1 chemistry combined with Dorado SUP basecalling has improved modal accuracies to Q20 (> 99%). This reduction in stochastic noise is what enables the differentiation of true biological variants at 1% frequency from sequencing artifacts. However, our study highlights a ‘bioinformatic gap’: standard tools like TB-Profiler, while excellent for routine surveillance, utilize default filters (e.g., BCFtools) that are optimized for purity rather than heteroresistance, effectively masking variants below 25% in our dataset. For clinical applications requiring high sensitivity for minor resistant clones, move-away-from-default ‘consensus’ calling toward deep-coverage pile-up analysis or specialized low-frequency variant callers is essential to avoid underestimating the true prevalence of heteroresistance.

Portable MinION Mk1B or PromethION P2 devices, without mandatory BSL-3 infrastructure, align with on-site sequencing initiatives in high-burden regions^38^. Integrating deep amplicons (LoD ≈ 1%) and AS (LoD ≈ 5%) on the Nanopore platform positions portable genomics as a decisive tool for closing the gap between molecular speed and phenotypic sensitivity in HR detection. Lowering the LoD to 1% for rifampicin/isoniazid and to 5% for emerging markers such as mmpR5 suggests that, supported by expanding mutation catalogues and robust low-frequency callers, this technology can progressively replace culture as the reference standard. This aligns with other studies demonstrating that deep sequencing can reveal subpopulations undetectable by standard molecular tests^43^.

This observation has important diagnostic and epidemiological implications. Standard molecular diagnostics often struggle to identify low-frequency variants; for instance, GeneXpert Ultra and LPAs may fail to report resistance when the mutant subpopulation is below 20% or 5%, respectively^17,18^. This diagnostic gap can lead to false susceptibility profiles in heteroresistant samples, whereas our nanopore-based approach pushes this limit down to 1%. Misclassification may result in inappropriate therapy, poor clinical outcomes, and continued transmission of resistant subpopulations^5^. Our findings therefore highlight the need for more sensitive diagnostic approaches, particularly in countries with high TB incidence and weak health systems.

While whole-genome sequencing (WGS) has traditionally relied on cultured isolates, recent advancements have enabled direct-from-sputum sequencing. A successful approach in this domain is whole-genome target enrichment using hybrid capture (e.g., RNA or dsDNA baits). This method physically enriches *M. tuberculosis* DNA from complex clinical samples prior to sequencing, overcoming the challenge of high host-DNA background. Recent studies and meta-analyses have demonstrated that hybrid capture can generate actionable, high-resolution genomic and resistance profiles directly from smear-positive sputum^44,45^. Furthermore, optimizing sample pre-processing—such as utilizing specific DNA thermo-protection buffers^46^ or enzymatic depletion of extracellular host DNA^47^—has been shown to significantly improve the pathogen-to-host DNA ratio. While these hybrid capture WGS methods provide comprehensive data, they often require extensive, multi-step library preparations, expensive probe sets, and are primarily optimized for short-read platforms like Illumina. In contrast, our study leverages Oxford Nanopore sequencing to assess two alternative strategies—amplicon sequencing and AS. These offer complementary advantages in turnaround time, portability, and, in the case of AS, computational enrichment without the need for additional physical hybridization steps.

The detection of heteroresistance carries potential implications for clinical management. Early identification of low-frequency resistant populations could allow for tailored treatment regimens that preempt the expansion of fully resistant clones. For instance, patients harboring low-frequency PZA-resistant variants might benefit from adjusted drug dosages or alternative regimens to reduce the risk of treatment failure. However, interpretation of these findings is complicated by the known limitations of phenotypic PZA susceptibility testing, which relies on acidic growth conditions and can yield variable results. This may hinder direct linkage between detected pncA mutations and phenotypic resistance. In this context, nanopore sequencing may serve as a complementary tool to conventional DST.

Furthermore, our approach could be expanded to other resistance-associated loci (*rpoB*, *katG*, *embB*, *gyrA*), enabling broad-spectrum resistance surveillance in real time. It could also be applied to extrapulmonary TB or pediatric cases, where bacterial loads are often insufficient for culture. Additionally, longitudinal studies could explore whether baseline heteroresistance correlates with treatment outcomes, relapse, or the emergence of multidrug resistance.

Despite its advantages, our study has limitations. First, our design utilized multiple DNA extraction methods, library preparation strategies, and sample types to optimize each workflow for its specific application. Direct comparisons between amplicon sequencing and adaptive sampling are complicated by differences in input material (whole-cell mixtures vs. purified DNA), fragment size, and background composition (sputum matrix vs. clean matrix). Second, the pilot nature of the workflow optimization involved a focused set of clinical specimens (12 sputum and 12 culture samples), meaning further studies with larger cohorts are needed to generalize these findings across diverse bacillary loads. Third, as is inherent to targeted approaches, amplicon sequencing is restricted to predefined loci, meaning resistance driven by novel or non-targeted genomic regions would not be captured. Finally, the number of clinical specimens analyzed directly in the workflow-optimization arm was modest, and the analytical limit-of-detection estimates—particularly for adaptive sampling—were derived from controlled synthetic mixtures rather than from a large clinical validation set. Consequently, the findings may not fully generalize to the broader range of sputum characteristics, bacillary loads, and host-background composition encountered in routine practice. Although the ultimate goal of these ONT workflows is to enable rapid, direct-from-sputum diagnostics, our study highlights that phenotypic culture remains the essential gold standard for validating these molecular thresholds and confirming low-frequency variants during assay development.

In conclusion, we provide evidence that nanopore-based amplicon sequencing with AS enables rapid and sensitive detection of heteroresistance in *M. tuberculosis*.

Our study suggests a hybrid algorithm: (i) Nanopore amplicons as a rapid rule-in when bacillary load is moderate-to-low so we have less DNA for detection; (ii) AS when the amount of DNA is high or full genomic surveillance is required; and (iii) culture back-up only for discrepancies or suspected HR < 1%. This pathway balances sensitivity, breadth and operational feasibility, echoing WHO guidance on rapid molecular tests followed by phenotypic confirmation.

This approach could significantly enhance TB diagnostics in resource-limited settings by identifying minority resistant variants directly from cultured clinical samples. Integrating this strategy into diagnostic and surveillance programs may improve therapeutic outcomes and reduce the spread of drug resistance in endemic regions. In addition, studying the detection of heteroresistance directly from clinical sputum samples could lead to new insights and improved diagnostic strategies.

## Methods

### Study design and sample collection

Our study was conducted in two main stages: workflow optimization and limit of detection (LoD) assessment. For workflow optimization, to establish sequencing performance for direct-from-sample analysis, we utilized clinical sputum samples from patients with pulmonary tuberculosis alongside cultured isolates. For the LoD assessment, to establish the limits of detection for heteroresistance, we evaluated our sequencing strategies using two distinct models: Amplicon sequencing and adapting sampling (AS). Amplicon sequencing was performed on synthetic whole-cell mixtures of susceptible (H37Rv) and resistant (DM97) reference strains, which were spiked into a TB-negative clinical sputum matrix. AS was performed on purified, high-molecular-weight DNA mixtures of the same reference strains. Clinical sputum samples were collected from patients undergoing TB screening at two sites in Peru: The Regional Hospital of Loreto (for smear-positive cases) and DIRESA Callao (for smear-negative cases), both endemic regions with high TB incidence. Samples were collected from September 2023 to August 2024.The samples were selected based on previous genotypic or phenotypic drug resistance testing. Sputum specimens (≥ 2 mL) were residual diagnostic material, transported and stored at 4 °C under standard biosafety conditions.

For the initial phase of workflow optimization, specifically to compare DNA extraction methods, a total of 33 anonymized residual diagnostic sputum samples were analyzed. As these were anonymized specimens, no patient demographic or clinical metadata were available, and the only recorded parameter was smear microscopy bacillary load. Following this extraction comparison, a representative subset of 12 smear-positive samples, together with 12 culture samples, was selected for subsequent workflow optimization, including evaluation of decontamination methods, DNA input (genomic DNA and PCR products), and library preparation on sequencing performance metrics.

For the limit of detection assessment using controlled mixtures, two *M. tuberculosis* reference strains were used.: the drug-susceptible H37Rv and a multidrug-resistant Peruvian clinical isolate, DM97. H37Rv was chosen for its well-characterized drug-susceptibility profile, while DM97 served as a standardized resistant control for comparative analysis^48^. Both strains were maintained in cryopreservation and handled under biosafety level III protocols (Supplementary Fig. S1).

#### Ethical approval

for the use of human biological samples was obtained from the Institutional Ethics Committee of the Universidad Peruana Cayetano Heredia (approval code: CONSTANCIA-CIEI-344-33-23; approval date: August 14, 2023). All experiments were conducted in accordance with relevant guidelines and regulations. The requirement for informed consent was waived by the Institutional Ethics Committee for the use of anonymized residual diagnostic sputum samples.

### Sputum decontamination and culture conditions

During workflow optimization, the clinical sputum samples were decontaminated using a saponin-based method^49^. Approximately 2 mL of each sputum sample were homogenized using a vortex at 2,500 rpm for 1 min, then allowed to rest for 2 min. The homogenate was centrifuged at 13,000 rpm for 10 min. The supernatant was discarded, and the pellet was resuspended in 625 µL of phosphate-buffered saline (PBS). Next, 500 µL of saponin were added (at 5% final concentration), and the mixture was incubated at room temperature for 10 min with gentle mixing every 3 min. Then, 200 µL of water and 30 µL of 5 M NaCl were added to denature proteins and neutralize the negative charge of human DNA. The homogenate was centrifuged at 8,000 rpm for 5 min, and then the supernatant was discarded. The pellet was washed three times with 800 µL of PBS by centrifugation at 8,000 rpm for 3 min each. The final pellet was resuspended in 400 µL of PBS and divided into two 200 µL aliquots for DNA extraction. No enzymatic digestion step was included in this host-DNA depletion workflow. Aliquots of this decontaminated pellet were also cultured on Middlebrook 7H11 medium at 37 °C for up to 5 weeks until visible colonies appeared.

For the limit of detection assessment, heteroresistant mixtures were prepared from the H37Rv and DM97 reference strains, which were thawed and subcultured in Middlebrook 7H9 broth. The heteroresistant mixtures were mixed with decontaminated smear-negative sputum. Prior to this phase, an internal comparison between the Saponin and NALC-NaOH protocols showed that, although Saponin yielded higher total DNA concentrations, both methods produced comparable downstream PCR amplification. Because equivalent amplification was the primary requirement for our sequencing assays, the standardized NALC-NaOH protocol was selected for the limit of detection assessment due to its simpler workflow. The smear-negative sputa were decontaminated using the standard N-acetyl-L-cysteine/sodium hydroxide (NALC–NaOH) method^20^. Equal volumes of sputum and 0.5% NALC–NaOH solution were mixed and incubated for 15 min with intermittent agitation, then neutralized with PBS and centrifuged at 3,000 rpm for 15 min. The resulting pellet was resuspended in 1.6 ml of PBS. Aliquots of the pellet were cultured on Middlebrook 7H11 agar medium at 37 °C for up to 5 weeks until visible colonies appeared.

### DNA extraction and quality assessment

Efficient and high-quality DNA extraction is essential for successful sequencing, especially in direct-from-sample workflows.

For workflow optimization, two DNA extraction methods based on different purification assessments were compared. We aimed to maximize the yield and integrity of *M. tuberculosis* DNA while minimizing host and contaminant DNA. The first method, a cost-effective in-house protocol referred to as Phenol, employed a phenol–chloroform–isoamyl alcohol extraction combined with mechanical lysis and magnetic AMPure XP bead cleanup (Beckman Coulter). While highly effective at maximizing total DNA recovery, this method is labor-intensive as it requires careful manual recovery of the supernatant and additional time for bead magnetization. In this protocol, 200 µL of decontaminated sample was mixed 1:1 with phenol: chloroform: isoamyl alcohol, then subjected to bead-beating using a FastPrep-24™ homogenizer and purified with AMPure XP beads (Beckman Coulter). DNA was finally eluted in 35 µL TE buffer. The second protocol, termed Kit, utilized the Quick-DNA™ Miniprep Plus kit (Zymo Research)^50^ with mechanical lysis. Samples were mixed with the kit’s BioFluid & Cell Buffer, lysed via the same bead-beating method, and then purified using spin columns, according to the manufacturer’s instructions, with a final elution volume of 35 µL, providing a simpler approach that reduced hands-on processing time by approximately half compared to the magnetic bead method. DNA concentration and purity were measured with a Qubit fluorometer (Thermo Fisher Scientific) and NanoDrop 2000 spectrophotometer (Thermo Fisher Scientific), and the fragment size distribution was assessed using a TapeStation analysis (Agilent Technologies).

For the limit of detection assessment, the choice of extraction method was based on the best-performing approaches identified during the workflow optimization phase. The Kit extraction was used for amplicons due to its improved amplification performance, sequencing output, and simpler workflow. In contrast, the Phenol method was retained for adaptive sampling (AS) to provide higher DNA yield and sufficient input mass for this approach, while maintaining fragment lengths compatible with efficient read-reject decisions.

### Primer design and generation

Targeted amplification of resistance-associated genes increases the sensitivity of variant detection and enables enrichment of low-abundance bacterial DNA.

During workflow optimization, fourteen genes associated with resistance to first-line drugs (rifampicin, isoniazid, pyrazinamide, and ethambutol) and second-line drugs (fluoroquinolones, ethionamide, and aminoglycosides) were selected for PCR amplification; specific gene-drug associations are detailed in Supplementary Table S2. Primers for these targets, adapted from previously published studies^51^, were organized into three primer pools according to gene size (see Supplementary Table S2). PCR was performed using Phusion High-Fidelity DNA Polymerase (Thermo Fisher Scientific), following the manufacturer’s instructions. Each PCR reaction (25 µL total volume) contained 50 ng of template DNA, 12.5 µL of 2× Phusion^®^ High-Fidelity PCR Master Mix, 0.75 µL DMSO, 3.5 µL of the appropriate primer pool (with each primer at 0.06–0.8 µM final concentration), and nuclease-free water to reach the final volume. Thermal cycling conditions included initial denaturation at 98 °C for 30 s, followed by 30 cycles of denaturation (98 °C, 10 s), annealing (62 °C, 20 s), and extension (72 °C, 30 s), with a final extension at 72 °C for 5 min.

For the limit of detection assessment, primer pool A (see Supplementary Table S3), composed of four primer pairs *(mabA-inhA*,* rpoB*,* pncA*, and *katG*), was used to amplify resistance genes for isoniazid, rifampicin, and pyrazinamide. The *rpoB* primers, both forward and reverse, were redesigned, now producing a fragment of 857 bp. For pool A, the same PCR conditions as described in the previous paragraph were used.

Amplification products (amplicons) were confirmed by electrophoresis on 0.8% agarose gels (96 V), then purified using a DNA Clean & Concentrator^®^-5 kit (Zymo Research) and eluted in 15 µL of elution buffer. Additionally, we performed a series of template DNA dilutions (from 10 ng/µL down to a 10^− 5^ dilution) to determine the minimum DNA concentration required for robust amplification.

### Determination of the limit of detection for heteroresistance using heteroresistant mixtures

During the limit of detection assessment, two targeted sequencing enrichment methods were compared: Amplicon sequencing and AS using heteroresistant mixtures of H37Rv and DM97 *M. tuberculosis* strains with susceptible and resistant profiles, respectively. Heteroresistant mixtures were generated to validate the detection of heteroresistance and establish sensitivity thresholds. In the amplicon sequencing, *M. tuberculosis* strains mixture was used and in the AS, DNA mixture from the same strains was used. This step ensured controlled conditions for benchmarking the detection of low-frequency variants.

*Amplicon sequencing using M. tuberculosis strains mixture.* A bacterial suspension in 0.04% Tween with OD of 0.2, equivalent to 1 × 10⁶ cfu/mL was prepared. Heteroresistant strain mixtures were prepared at defined H37Rv: DM97 ratios of 100:0, 99:1, 95:5, 90:10, 75:25, 50:50, and 0:100. Aliquots (50 µL) of each mixture were spiked into 150 µL of decontaminated TB-negative sputum samples and, in parallel, into PBS as a matrix-free control. DNA was extracted using the Kit method. DNA concentrations were measured using Qubit (Thermo Fisher Scientific). Genomic DNA from each confirmed mixture was then extracted and processed for nanopore sequencing as described below.

*Phenotypic Drug Susceptibility Testing (DST) as ground truth.* To validate the heteroresistant ratios and establish phenotypic sensitivity thresholds, the presence of resistant subpopulations in the synthetic mixtures was confirmed using the agar proportion method. Mixtures were plated onto 7H11 agar quadrant plates supplemented with 5% OADC, 0.2% glycerol, 0.05% Tween-80, and contamination-selective antibiotics (polymyxin B, amphotericin B, carbenicillin, and trimethoprim). The quadrants contained isoniazid (0.4 µg/mL), rifampicin (1 µg/mL), pyrazinamide (100 µg/mL), or no antibiotic (control). To ensure pyrazinamide activity, the agar for the PZA quadrant was adjusted to an acidic pH of 5.8, and an additional control quadrant without PZA at pH 5.8 was also included The undiluted mixture and two serial dilutions (10^− 1^ and 10^− 2^) were plated using 30 µL volumes (distributed as three 10 µL drops per quadrant), and each sample was plated in triplicate. Inoculated plates were incubated at 37 °C for up to five weeks. Resistance was analyzed by calculating the proportion of resistant bacilli: (CFU in drug-containing quadrant / CFU in control quadrant) × 100, with a threshold of ≥ 1% defining phenotypic resistance. After which colony counts were used to determine the lowest proportion of resistant bacteria detectable by culture. Because the colonies at the lowest proportions (1% and 5%) were extremely small and sparse, an inverted microscope was required to accurately observe and count them across the drug quadrants. It should be noted that the photographs shown in Supplementary Fig. S8 are representative plates of the 10^− 1^ dilution, provided primarily to illustrate the relative increase in resistant colonies. Due to their size, the microcolonies present at the 1% and 5% limits are not easily discernible to the naked eye in these representative images. Genomic DNA from each confirmed mixture was then extracted and processed for nanopore sequencing as described below.

*AS using M. tuberculosis DNA mixture.* AS was applied exclusively to purified DNA mixtures. This approach allowed us to isolate and quantify the method’s intrinsic sensitivity for detecting low-frequency resistant subpopulations under controlled, culture-like conditions. DNA from each H37Rv and DM97 were extracted by phenol–chloroform. The extracted DNA underwent quality control by TapeStation to assess fragment size, then was mildly sheared using G-tubes at 12,000 rpm to achieve an approximate N50 of ~ 10 kb. DNA concentrations were measured with a Qubit fluorometer, and mixtures were prepared by combining H37Rv and DM97 DNA in defined ratios of 100:0, 99:1, 95:5, 90:10, 75:25, 50:50, and 0:100 aiming for a total of ~ 500 ng in 12 µL. Each DNA mixture was then processed according to the nanopore sequencing workflow described below.

Each mixture was prepared in three biological replicates for both amplicon and AS/standard non-enriched nanopore WGS experiments.

### Library preparation and barcode multiplexing

During workflow optimization, DNA libraries were prepared using the Ligation Sequencing Kit (SQK-NBD114) and Rapid Barcoding Kit (SQK-RBK114) (Oxford Nanopore Technologies) according to the manufacturer’s protocol. Native barcodes were used for multiplexing, allowing up to 12 samples per run. Ligation of sequencing adapters and barcodes was followed by AMPure bead purification.

*Rapid Barcoding protocol.* For genomic DNA libraries, approximately 250 ng of genomic DNA was brought to 9 µL with nuclease-free water, then enzymatically fragmented and barcoded via transposase tagmentation (incubation at 30 °C for 2 min and 80 °C for 2 min; no PCR amplification cycles were used). For amplicon libraries, an input of 50 ng of pooled PCR products were processed using the same rapid barcoding steps.

*Ligation Native Barcoding protocol.* For genomic DNA libraries, approximately 400 ng of starting material underwent end-repair, enzymatic barcode ligation, and adapter attachment according to the kit instructions. Alternatively, for amplicon libraries, a specific input of 150 ng of amplicon DNA was used (the FFPE DNA repair step was omitted as it was not required).

To normalize library loading across all samples, the final libraries were quantified using Qubit, and fragment size distributions were evaluated on an Agilent TapeStation to calculate molarity. Approximately 75 fmol in 11 µL of each prepared library was loaded onto a MinION flow cell (R10.4.1). The sequencing runs were performed with MinKNOW software (v23.07.5).

For the limit of detection assessment, DNA libraries were prepared using Ligation Native Barcoding protocol, and for AS run, the feature was enabled in MinKNOW to target specific genomic regions.

### Nanopore sequencing with AS

To achieve high coverage of target regions from synthetic heteroresistant mixtures, we used Oxford Nanopore’s AS, a real-time enrichment strategy. This allowed selective sequencing of AMR genes and adjacent regions while depleting non-target DNA. For targeted sequencing via AS, high–molecular weight genomic DNA from H37Rv and DM97 was sheared to an ~ 10 kb fragment length (N50). This library was prepared using a ligation-based workflow and sequenced on the PromethION P2 with a R10.4.1 flow cell to increase throughput.

AS was configured using MinKNOW software (Oxford Nanopore Technologies), providing a target reference FASTA file containing AMR genes. It used a BED file that defined 14 known resistance-associated genes (*rpoB*,* katG*,* inhA–mabA*,* pncA*,* rpsL*,* gyrA*,* gyrB*,* embB*,* embC–embA*,* ethA*,* oxyR–ahpC*,* mmpR5*,* eis*, and *rrs*) along with 10 kb of flanking sequence on each side of each gene. The BED file also included the direct repeat (DR) region for spoligotyping, to facilitate strain genotyping during sequencing. During the AS run, off-target reads (e.g., non-AMR DNA from TB) were actively ejected to enrich for the specified regions. For internal sequencing configuration, approximately half of the nanopores were allocated to AS mode, while the other half of the flow cell was operated under the standard whole-genome sequencing (WGS) configuration. This mixed setup enabled simultaneous targeted enrichment and standard sequencing within the same run. To maintain performance over the duration of this long sequencing run, nuclease flushes were performed approximately every 20 h to reduce pore blockage and sustain throughput. The run lasted 48 h. Sequencing progress was monitored in real time to ensure sufficient coverage of the target regions.

### Bioinformatic and statistical analysis

*Limit of detection and validation.* To evaluate the sensitivity of the method for detecting heteroresistant subpopulations, we analyzed the synthetic DNA mixtures. The aim was to determine the minimum detectable allele frequency and compare it to the background noise of the sequencing platform. Findings were validated by cross-checking with control samples containing only wild-type or mutant alleles. Correspondingly, 100:0 H37Rv mixtures were used as blanks for AS and for amplicon experiments (with and without sputum), while 0:100 DM97 mixtures served as positive controls.

Nonparametric estimates of the limit of blank (LoB) and LoD were calculated following CLSI EP17-A2 recommendations^52^. For each sequencing method, LoB was defined as the 95th percentile of allele frequencies observed in blank samples (AF_expected = 0). To estimate the LoD, we examined increasing levels of expected allele frequencies (AF_expected > 0) and computed the empirical detection probability, defined as the proportion of replicates with observed AF exceeding the LoB. The LoD was assigned as the lowest expected allele frequency at which ≥ 95% of replicates were detected.

*Data Processing and Quality Control.* Raw signal data from the Nanopore runs was basecalled using Dorado v0.7.3^53^ with the super accuracy model (dna_r10.4.1_e8.2_400bps_sup). The base called reads were then subjected to additional filtering with MinKNOW: adapter sequences were trimmed, reads with average quality score < 9 were discarded, and reads shorter than 200 bp were removed. Read quality metrics, including read length N50, total bases generated, and median Q-score, were compiled using NanoPlot v1.41.6^54^ for each dataset.

*Taxonomic Classification and Read Mapping.* For clinical sputum samples, host DNA was identified and discarded by mapping reads to the human reference genome (GRCh38) using minimap2^55^. The remaining non-human reads were then taxonomically classified using Kraken 2 (Standard database, release 2024-09-04), and only reads explicitly assigned to the *M. tuberculosis complex* were extracted for downstream analysis. Conversely, for cultured isolates, purified DNA mixtures, and amplicons where contamination was negligible, reads were mapped directly to the *M. tuberculosis* H37Rv reference genome using minimap2. In both workflows, per-gene coverage across the targeted resistance loci was calculated using Mosdepth^56^.

*Normalization of Sequencing Depth.* For the targeted four-gene amplicon panel, a direct comparison of sequencing depth between the Phenol-Ligation and Phenol-Rapid library preparation methods, presented in Table 1, was confounded by the use of separate MinION flow cells with substantially different performance characteristics. Specifically, the two runs varied greatly in their number of starting active pores and total run duration. To isolate the intrinsic efficiency of each library preparation protocol from these experimental variables and enable a valid comparison, the mean coverage depth for each target gene was normalized. This metric, expressed as depth per 1,000 pore-hours (× kPH⁻¹), was calculated by multiplying the mean sequencing depth by 1,000, and then dividing this by the product of the starting active pores and the run duration in hours.

DM97 characterization. A subset of barcodes containing only the DM97 isolate was selected for strain characterization. The FASTQ files were merged and a de novo assembly of the DM97 strain was generated using Flye (v2.9.5). Assembly curation was performed based on circularization and contig length. Quality metrics of the curated genome were evaluated with CheckM2 (v1.1.0). TB-Profiler (v6.6.0)^57^ was used to determine the spoligotype patterns for lineage assignment, and to identify four resistance-associated variants (Table 2).

Variant Calling and Resistance Profiling. Those resistance gene loci were subsequently specified in a BED file. To benchmark detection sensitivity, we analyzed synthetic heteroresistant mixtures spanning resistant allele frequencies of 0%, 1%, 5%, 10%, 25%, 50%, and 100%, and compared calls obtained by direct pileup inspection with those generated by TB-Profiler (bcftools v1.22) using the default settings. Direct pileup inspection was used as a baseline since it allows manual verification of low-frequency variants, providing a more sensitive reference than automated calling. Allele frequencies reported by each method were compared against the known mixing ratios to characterize method-specific limits of detection and assess accuracy and sensitivity for low-frequency mutations in mixed-infection scenarios. Finally, we evaluated two sequencing workflows, amplicon sequencing and AS, by comparing the concordance between expected and observed allele frequencies.

### Statistical Analysis and Visualization

All statistical analyses and plotting were performed in R (RStudio). Coverage depth and allelic frequency for each resistance gene were calculated for all samples, and results were visualized using the *ggplot2* and *dplyr* packages. For AS runs, we extracted read counts from the MinKNOW log files to quantify the reads classified as on-target (AS_Pass) versus those that were rejected (AS_Block).

## Electronic Supplementary Material

Below is the link to the electronic supplementary material.

Supplementary Material 1

## Data Availability

To ensure reproducibility and scalability, FASTQ files are deposited in the NCBI Sequence Read Archive under BioProject accession number PRJNA1314197.

## Code availability

To ensure reproducibility and scalability, the entire bioinformatics analysis was implemented in bash and Nextflow, scripts and code are available at GitHub (https://github.com/omar-romero-r/TB-Heteroresistance).
