# Supplementary material for: Detection of heteroresistance in Mycobacterium tuberculosis using nanopore-based amplicon sequencing and adaptive sampling
Source: Sci Rep. 2026 Jun 4;16:25566. doi: 10.1038/s41598-026-53258-0 (PMC13478595; doi:10.1038/s41598-026-53258-0)
Supplement: Supplementary file 1 [file 41598_2026_53258_MOESM1_ESM.docx]

# **Supplementary Figures**

**Supplementary Figure S1.** Study workflow scheme. The top panel (Workflow Optimization) illustrates the comparison of DNA extraction and library preparation methods for sputum and culture samples, using WGS and amplicons. The bottom panel (Limit of Detection) details the enrichment strategies and synthetic mixtures used to determine sensitivity thresholds.

**Supplementary Figure S2**. Quality control of DNA extracted from saponin-decontaminated sputum samples using two different methods.

**(S2a)** DNA concentration and purity ratios obtained from two extraction methods: Phenol (Phenol-Chloroform extraction with FastPrep lysis and AMPure bead purification) and Kit (Commercial Zymo kit with FastPrep lysis and column purification).

**(S2b)** Distribution of DNA fragment peak size measured by automated electrophoresis for samples extracted using the Kit and Phenol methods. Points represent individual samples and boxplots indicate the median and interquartile range. Mean peak size was 15.8 ± 1.8 kb for Kit and 7.5 ± 0.7 kb for Phenol.

**Supplementary Figure S3**. Agarose gel electrophoresis of 14 amplicons generated from clinical or cultured samples using three primer pools.
 PCR amplification targeted 14 genomic regions grouped into three primer pools:
 Pool A: *rpoB* (3662 bp), *inhA* (2902 bp), *katG* (2503 bp), *ethA* (1828 bp), *rpsL* (939 bp);
 Pool B: *embB* (3636 bp), *gyrB* (2380 bp), *rrs* (1927 bp), *oxyR* (1138 bp), *pncA* (876 bp);
 Pool C: *gyrA* (2978 bp), *embC* (1652 bp), *eis* (1329 bp), *mmpR5* (959 bp).
 **(S3a)** Cultured samples extracted with the Phenol method. The brightly fluorescent material retained in the loading wells corresponds to abundant, high-molecular-weight genomic DNA template that can fail to migrate into the gel.
 **(S3b)** Cultured samples extracted with the Kit method.
 **(S3c)** Sputum samples extracted with the Phenol method, showing nonspecific amplification patterns characteristic of clinical material.
 Each set of three lanes per sample corresponds to Pools A, B, and C, respectively, for a total of 12 samples.

**Supplementary Figure S4**. Boxplots comparing the number of *M. tuberculosis* reads (log-scale) obtained from metagenomic sputum samples under each processing method (library prep × DNA extraction)

**Supplementary Figure S5.** Coverage depth across antimicrobial resistance (AMR) genes, stratified by DNA extraction methods (14-gene panel).

**Supplementary Figure S6.** Relationship between gene length and sequencing depth for sputum samples processed by phenol extraction and ligation library preparation (14-gene panel).

**Supplementary Figure S7.** Scatter plot with a linear regression line showing the relationship between gene length and sequencing coverage depth in culture TB samples using nanopore amplicon sequencing. Gene amplicons were processed by the ligation method; each data point represents a gene and is colored according to gene category/type.

**Supplementary Figure S8**. Representative plates of the agar proportion method for the determination of resistance to rifampicin, isoniazid and pyrazinamide. Seven mixtures of H37Rv and DM97 (0 to 100%) were used to determine the percentage of resistance of *M. tuberculosis.* The 0% DM97 condition corresponds to a 100% H37Rv drug-susceptible control and demonstrates inhibition in the antibiotic-containing quadrants*. Note: Because these are representative macroscopic images, the microcolonies present at the lowest resistance proportions (1% and 5%) are not readily visible to the naked eye and required an inverted microscope for accurate quantification.*

.

**Supplementary Figure S9.** Genome‑wide sequencing depth profile obtained during Nanopore adaptive sampling. The x‑axis represents chromosomal position and the y‑axis the read depth.

**Supplementary Figure S10.** Comparison of resistant allele detection between pileup inspection and TB-Profiler (using the variant caller bcftools v1.22) across synthetic heteroresistant mixtures. Direct pileup inspection was able to recover resistant subpopulations down to ~1%, whereas TB-Profiler (bcftools) consistently failed to identify variants below 25% frequency, highlighting its limited sensitivity for low-frequency resistance alleles.

| Sample ID | DNA concentration (ng/µL) | | DNA yield (ng) | | 260/280 | | 260/230 | |
| --- | --- | --- | --- | --- | --- | --- | --- | --- |
|  | Phenol | Kit | Phenol | Kit | Phenol | Kit | Phenol | Kit |
| 1 | 604 | 59.2 | 30200 | 2960 | 1.89 | 1.88 | 2.32 | 2.13 |
| 2 | 342 | 101 | 17100 | 5050 | 2.03 | 1.89 | 2.27 | 2 |
| 3 | 212 | 111 | 10600 | 5550 | 2.05 | 1.87 | 2.21 | 1.87 |
| 4 | 121 | 52.4 | 6050 | 2620 | 1.88 | 1.84 | 2.13 | 2 |
| 5 | 158 | 63.2 | 7900 | 3160 | 1.83 | 1.86 | 1.9 | 2.3 |
| 6 | 47 | 31.6 | 2350 | 1580 | 1.93 | 1.93 | 2.04 | 1.72 |
| 7 | 168 | 86.4 | 8400 | 4320 | 1.87 | 1.86 | 1.95 | 2.26 |
| 8 | 118 | 134 | 5900 | 6700 | 2.01 | 1.88 | 2.2 | 2.27 |
| 9 | 4.64 | 14.3 | 232 | 715 | 1.95 | 1.89 | 1.07 | 1.73 |
| 10 | 73.6 | 58.6 | 3680 | 2930 | 1.96 | 1.91 | 2.03 | 1.77 |
| 11 | 16.1 | 22 | 805 | 1100 | 1.94 | 1.93 | 1.7 | 1.65 |
| 12 | 0.707 | 0.362 | 35.35 | 18.1 | 2.2 | 1.91 | 3 | 0.31 |
| 13 | 34.4 | 45.2 | 1720 | 2260 | 2 | 1.92 | 2.15 | 2.05 |
| 14 | 24.8 | 20.2 | 1240 | 1010 | 1.71 | 1.79 | 1.72 | 1.94 |
| 15 | 173 | 102 | 8650 | 5100 | 1.8 | 1.87 | 1.7 | 2.3 |
| 16 | 82.8 | 120 | 4140 | 6000 | 1.9 | 1.89 | 1.72 | 2.18 |
| 17 | 412 | 108 | 20600 | 5400 | 1.75 | 1.86 | 1.68 | 2.29 |
| 18 | 1148 | 175 | 57400 | 8750 | 1.8 | 1.86 | 1.82 | 2.27 |
| 19 | 99.6 | 116 | 4980 | 5800 | 1.59 | 1.86 | 1.6 | 0.36 |
| 20 | 107.2 | 62.6 | 5360 | 3130 | 1.71 | 1.88 | 1.72 | 2.27 |
| 21 | 35.2 | 28.8 | 1760 | 1440 | 1.6 | 1.85 | 1.59 | 2.08 |
| 22 | 117.2 | 80.6 | 5860 | 4030 | 1.71 | 1.86 | 1.6 | 2.27 |
| 23 | 1168 | 220 | 58400 | 11000 | 1.9 | 1.88 | 2.06 | 2.21 |
| 24 | 28.2 | 30.2 | 1410 | 1510 | 1.59 | 1.87 | 1.56 | 1.9 |
| 25 | 45.8 | 75.8 | 2290 | 3790 | 1.88 | 1.85 | 1.85 | 1.88 |
| 26 | 120 | 102 | 6000 | 5100 | 1.92 | 1.87 | 1.87 | 1.92 |
| 27 | 62.4 | 62.4 | 3120 | 3120 | 1.89 | 1.86 | 1.86 | 1.89 |
| 28 | 30.4 | 74.8 | 1520 | 3740 | 1.88 | 1.86 | 1.86 | 1.88 |
| 29 | 32.2 | 52.2 | 1610 | 2610 | 1.83 | 1.89 | 1.89 | 1.83 |
| 30 | 86.7 | 65.2 | 4335 | 3260 | 1.88 | 1.86 | 1.86 | 1.88 |
| 31 | 124 | 68.9 | 6200 | 3445 | 1.83 | 1.85 | 1.79 | 2.1 |
| 32 | 264 | 84.5 | 13200 | 4225 | 1.89 | 1.86 | 1.84 | 2.0 |
| 33 | 79 | 64.3 | 3950 | 3215 | 1.85 | 1.87 | 1.86 | 1.98 |

## **Supplementary Table S1** . Concentration, DNA yield, and quality ratios of 33 sputum samples using the phenol method and a kit.

| Pool | Gene | Size (bp) | Targeted Drug(s) | Forward primer (5′ - 3′) | Reverse primer (5′ - 3′) |
| --- | --- | --- | --- | --- | --- |
| *1* | *mabA- inhA* | 2902 | Isoniazid, Ethionamide | AATGGAAATCGACTGGTCAGGT | CTTGTAATCGCAACTCGTGGTC |
|  | *rpoB* | 3662 | Rifampicin | CTCCTCTAAGGGCTCTCGTT | GCGGTAGCAAGACCGATG |
|  | *ethA* | 1828 | Ethionamide | GACGGCCTCGACATTACGTT | TTGCTGAAGTCGGTGCTCTC |
|  | *katG* | 2503 | Isoniazid | GGACCATAACGGCTTCCTGT | CATTTCGGCGCCCTTTCTC |
|  | *rpsL* | 939 | Streptomycin | AATACACCGCTGCGACTAGG | GACCAACTGCGATCCGTAGA |
| *2* | *embB* | 3636 | Ethambutol | GATCGGTGGAGCAGTACCA | GCTGAGGAGACTTTTGTGGACTG |
|  | *gyrB* | 2380 | Fluoroquinolones | GCGCGGTTAGATGGGTAAA | AAGCCGGAATCGAACATTG |
|  | *rrs* | 1927 | Amikacin, Kanamycin, Capreomycin | AGATCGAACGGGTATGCTGTTA | GGCCAACTTTGTTGTCATGC |
|  | *oxyR-*  *ahpC* | 1138 | Isoniazid | GTGGTTGCTATGCGATCAGG | GTCGAGCACTCGCAGTACCT |
|  | *pncA* | 876 | Pyrazinamide | GACGGATTTGTCGCTCACTA | CCCGATGAAGGTGTCGTAGAA |
| *3* | *mmpR5* | 959 | Bedaquiline, Clofazimine | ATCAGTCGTCCTCTCCGGT | GCTCGTCCTTCACTTCGCC |
|  | *eis* | 1329 | Kanamycin | CCGGTACTTGCTCTGCAC | CGAAGCAGCTGGGAATCTTT |
|  | *embC-*  *embA* | 1652 | Ethambutol | ACAGCGGTTGACGCCTTACT | GTACTTGATGCGTGCCGATT |
|  | *gyrA* | 2978 | Fluoroquinolones | CGCCGCTGTACAAACTCAAG | CCCGACTCCTAACACTCGTA |

## **Supplementary Table S2** . Fourteen primers used to amplify *M. tuberculosis* genes.

| Pool | Gene | Size (bp) | Forward primer (5′ - 3′) | Reverse primer (5′ - 3′) |
| --- | --- | --- | --- | --- |
| A | *mabA - inhA* | 2902 | AATGGAAATCGACTGGTCAGGT | CTTGTAATCGCAACTCGTGGTC |
|  | *rpoB* | 857 | GCGCAGACGCTGTTGG | TCCACCTCAGACGAGGGC |
|  | *pncA* | 876 | GAC GGA TTT GCT GCT CTA | CCCGATGAAGGTGTCGTAGAA |
|  | *katG* | 2503 | GGACCATAACGGCTTCCTGT | CATTTCGGCGCCCTTTCTC |

## **Supplementary Table S3** . Four primers used to amplify *M. tuberculosis* genes.
